# Midostaurin added to 10-day decitabine, for patients unfit for intensive chemotherapy with AML and higher risk MDS, irrespective of *FLT3* mutational status, does not improve outcome

**DOI:** 10.1007/s00277-024-06033-y

**Published:** 2024-10-05

**Authors:** Gerwin Huls, Dana A. Chitu, Lidwine Tick, Rinske Boersma, Dimitri Breems, Alexandra Herbers, Saskia K. Klein, Suzan de Jonge, Peter E. Westerweel, Marjan Cruijsen, Mels Hoogendoorn, Marlous Cuijpers, Dries Deeren, Benjamin Bailly, Otto Visser, Anna van Rhenen, Eduard F. M. Posthuma, Peter J. M. Valk, Jacqueline Cloos, Emanuele Ammatuna, Jeannine M. Refos, R. Fakkert, Bob Löwenberg, Gert J. Ossenkoppele

**Affiliations:** 1 Department of Hematology, University Medical Center Groningen, PO Box 30.001, Groningen, 9700 RB The Netherlands; 2 Department of Hematology, Hemato Oncology Foundation for Adults in the Netherlands, Rotterdam, The Netherlands; 3 Department of Hematology, Maxima Medical Center, Veldhoven, The Netherlands; 4 Department of Hematology, Amphia Hospital, Breda, The Netherlands; 5 Department of Hematology, ZNA Stuivenberg/Middelheim, Antwerp, Belgium; 6 Department of Hematology, Jeroen Bosch Hospital, Den Bosch, The Netherlands; 7 Department of Hematology, Meander Hospital Amersfoort, Amersfoort, The Netherlands; 8 Department of Hematology, Canisius Wilhelmina Hospital, Nijmegen, The Netherlands; 9 Department of Hematology, Albert Schweitzer Hospital, Dordrecht, The Netherlands; 10 Department of Hematology, Catharine Hospital, Eindhoven, The Netherlands; 11 Department of Hematology, Medical Center Leeuwarden, Leeuwarden, The Netherlands; 12 Department of Hematology, Rijnstate Hospital, Arnhem, The Netherlands; 13 Department of Hematology, AZ Delta Roeselare, Roeselare, Belgium; 14 Department of Hematology, Hopital de Jolimont, Haine-Saint-Paul, Belgium; 15 Department of Hematology, Isala Hospital, Zwolle, The Netherlands; 16 Department of Hematology, University Medical Center Utrecht, Utrecht, The Netherlands; 17 Department of Hematology, Reinier de Graaf Hospital, Delft, The Netherlands; 18 Department of Hematology, Erasmus MC Cancer Center, Erasmus University Medical Center, Rotterdam, The Netherlands; 19 Department of Hematology, Amsterdam UMC, VU University Medical Center, Cancer Center Amsterdam, Amsterdam, The Netherlands

**Keywords:** Older, AML, Decitabine, Midostaurin, Phase II trial

## Abstract

The treatment of older patients with acute myeloid leukemia (AML) considered unfit for receiving intensive chemotherapy is challenging. Based on the hypothesis that addition of the broad tyrosine kinase inhibitor (TKI) midostaurin could improve the response to hypomethylating agents, irrespective of *FLT3* gene mutational status, we conducted a randomized phase II multicenter study to assess the tolerability and efficacy of the addition of midostaurin to a 10-day schedule of decitabine in unfit (i.e. Hematopoietic Cell Transplantation Comorbidity Index (HCT-CI) ≥ 3) AML and higher risk myelodysplasia (MDS) patients (HOVON155 trial). In total, 140 eligible patients were randomly (1:1) assigned to treatment with 10-days of decitabine alone (*N* = 70) or combined with midostaurin (50 mg bid;starting the day following the last dose of decitabine), (*N* = 70). Addition of midostaurin was well tolerated and the number of AEs was comparable for both treatment arms. Early death rates (< 30 days) were similar as well (10%). In the decitabine plus midostaurin arm 24% reached CR/CRi, the median OS was 4.8 months and 1-yrs OS was 31% which compared with 34% CR/CRi, median OS of 7.4 months and 1-yrs OS of 37% for the decitabine alone group (NS). Thus, while the addition of midostaurin appears safe, it does not enhance therapeutic efficacy of decitabine in unfit AML patients.

**Supplementary Information:**

The online version contains supplementary material available at 10.1007/s00277-024-06033-y.

## Key points

- Midostaurin added to 10-day decitabine does not improve response nor survival in patients with newly diagnosed, *FLT3* mutation agnostic, AML unsuitable for intensive chemotherapy.

**Supplementary Information:**

The online version contains supplementary material available at 10.1007/s00277-024-06033-y.

## Introduction

About 75% of AML patients are 60 years of age or older [1]. The treatment of older AML patients remains challenging. Treatment outcome is dependent on patient characteristics (e.g. age, performance, co-morbidities), disease characteristics (cytogenetic and molecular abnormalities, WBC count) and personal preference of the patient [2]. Hypomethylating agents (HMA), like azacitidine and decitabine, are particularly feasible in older patients and patients with co-morbidities. Recent phase 3 trials have shown the efficacy of azacitidine and decitabine compared with conventional care for older patients with AML [3–5]. Results of treatment with modifications of the standard azacitidine (seven days 75 mg/m^2^ SC; every four weeks) and decitabine (five days 20 mg/m^2^ IV; every four weeks) schedules have been reported. In a hallmark study, 52 newly diagnosed older AML patients were treated with the 10-day decitabine schedule, 21 patients (40%) achieved a CR and the median OS was 318 days [6]. The clinical value of the 10-day decitabine schedule is also supported by the recently reported data of a large phase III trial in older AML patients (≥ 60 years) which showed comparable survival (HR 1.04; 95% CI 0.86–1.26) between conventional intensive chemotherapy, based on cytarabine combined with an anthracycline (“3 + 7”), and the 10-day decitabine schedule, when followed by the consolidation option of an allogeneic hematopoietic cell transplantation [7].

It remains important to further improve the cumulative CR/CRi rates, survival and particularly reduce the relapse rate of HMA treatment. Some attempts to improve the efficacy of the HMA backbone have been successful. Recently, it has been shown, in prospective randomized trials, that azacitidine treatment in older patients unfit for intensive chemotherapy can be improved by the addition of venetoclax (HR 0.66; 95% CI 0.52–0.85) and by the addition of ivosidenib for those patients with mutated *IDH1* (HR 0.33; 95% CI 0.16–0.69) [8, 9]. Data obtained in a phase I study, which sequentially administered decitabine and midostaurin in newly diagnosed elderly or relapse /refractory adult patients with AML (13% with *FLT3* mutations), showed in the intention to treat analysis that 57% of patients achieved stable disease and 25% had a complete haematological response [10]. Stimulated by these data we hypothesized that midostaurin, a broad tyrosine kinase inhibitor, when added to decitabine, might improve outcome of decitabine treatment. Midostaurin is a multitargeted kinase inhibitor that inhibits several kinases, including protein kinase Cα (PKCα), vascular endothelial growth factor receptor 2 (VEGF2), KIT, platelet-derived growth factor receptor (PDGFR), and wild-type and/or mutant FLT3 tyrosine kinases [11]. Midostaurin has been approved for use in combination with intensive chemotherapy for younger and older adults with *FLT3* mutations [12, 13]. The addition of the more specific and potent FLT3 inhibitor gilteritinib to azacitidine in older unfit patients with AML with mutated *FLT3* (LACEWING trial) did not provide supportive evidence to improve OS [14].

Here we present the final analysis of the HOVON-155 study. In this phase II study, older patients (≥ 66 years) with AML or higher-risk MDS (IPSS-*R* > 4.5), including both wild type and mutated *FLT3* disease, considered not suitable for intensive cytotoxic treatment, were randomized between 10-day decitabine with or without midostaurin. The aim was to assess the value of adding midostaurin to 10-days of decitabine with respect to CR/CRi after 3 cycles of treatment (primary endpoint) and OS (secondary endpoint).

## Methods

### Study design, patients and treatment

This is a randomized phase II multicenter study (HOVON155; EudraCT number 2018-000047-31) (protocol is available at: www.hovon.nl). This study was approved by the Medical Ethical committee of the University Medical Centre Groningen, and all participants provided informed consent in accordance with the Declaration of Helsinki. Previously untreated adults who were ≥ 66 years old and not considered eligible for intensive chemotherapy, with AML, or with higher risk MDS (IPSS-*R* > 4.5), a World Health Organization (WHO) performance status of ≤ 2, and a written informed consent, were eligible. Patients were considered not suitable for intensive chemotherapy if they had co-morbidities that would put them at high risk for early death when treated with intensive chemotherapy (defined as hematopoietic cell transplantation co-morbidity index (HCT-CI) ≥ 3) or if the patient declined to receive intensive chemotherapy. The limit for HCT-CI ≥ 3 was chosen, because it has been shown that an HCT ≥ 3 is associated with an early mortality rate (< 30 days) of about 30%, in older patients with AML treated with intensive chemotherapy [15]. Based on the karyotype and molecular genotype of the leukemic cells, patients were classified into prognostic categories according to the European LeukemiaNet (ELN) 2017 criteria [16].

**Fig. 1:**
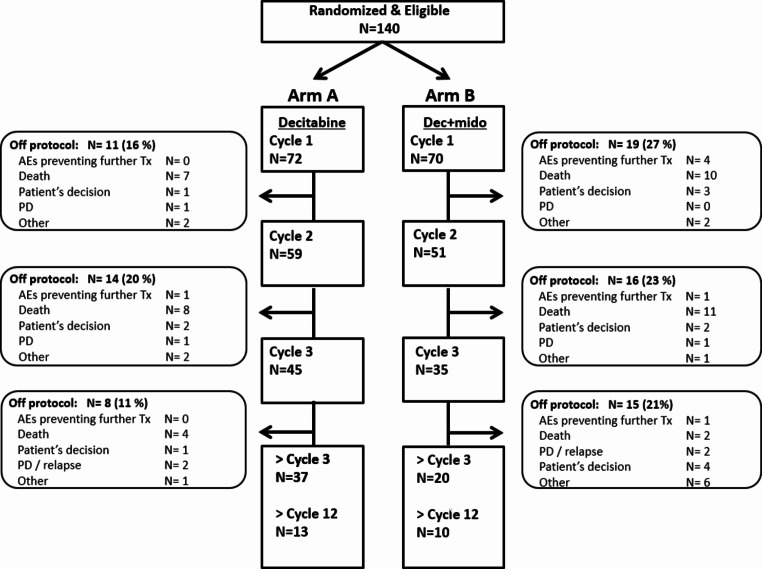
CONSORT study diagram. Arm A = 10-day decitabine; arm B = 10-day decitabine plus midostaurin. *AE* Adverse Event; *PD* Progressive Disease

Patients were randomly assigned (1:1) to 10-days of decitabine without (arm A) or with (arm B) midostaurin 50 mg bid (Fig. 1). In arm B midostaurin was given sequentially with decitabine, starting the day after the last dose of decitabine until the day before the start of the next cycle of decitabine. The duration of decitabine in the 2nd and 3rd cycle (i.e. 5 or 10 days) was dependent on the % of bone marrow blasts (i.e. <5% or ≥ 5%), as determined by cytomorphology, at the day-28 bone marrow examination (Fig. 1). Cycle 2 started within 1 week after day-28 bone marrow evaluation, independent of recovery of peripheral counts. Cycle 3 started if neutrophils were ≥ 0.5 × 10^9^/L or platelets were ≥ 25 × 10^9^/L with a maximum delay of 2 weeks. From cycle 4 onwards treatment could only be continued with 5-day decitabine (with or without midostaurin).

### Molecular analyses and flowcytometry

Details on the methods used for molecular analyses and flowcytometry are described in the supplementary data.

### Study endpoints and statistical analysis

The aim of this study was to decide whether the addition of midostaurin to standard induction treatment could be sufficiently effective to warrant continuation to a phase III study. The observed difference in CR/CRi rates (Dcr) between the investigational arm B and the standard arm A was used as criterion in the decision rules. At final analysis the decision to stop because of inefficacy (i.e. not to continue with phase III) was based on Dcr ≤ 0 or the upper-limit of the 80% confidence interval is < 25%.

The primary endpoint of the study was cumulative CR/CRi after 3 cycles (for all patients (MDS and AML). A patient was considered to have a CR/CRi if this response was attained within the first three induction cycles and the patient did not relapse on a consecutive cycle (within these three induction cycles). All other patients without “CR/CRi after 3 cycles” (including PR and morphologic leukemia free state (MLFS)) were classified as “induction failure”.

Secondary endpoints included safety, tolerability and efficacy profile (response rate (CR, CRi, MLFS, PR)), event free survival (EFS) and overall survival (OS)). Overall survival (OS) was defined as the time from the date of randomization to the date of death, whatever the cause. The follow-up of patients still alive was censored at the moment of last visit/contact. Event-free survival (EFS) was defined as the time from registration to induction failure, death or relapse, whichever occurred first. Kaplan–Meier survival curves and Cox regression tests were used to compare the survival distributions between the treatment arms.

## Results

### Study patients and treatment

From January 2nd 2020 until November 11th 2021 140 patients have been registered for the study. The database was closed on November 25th 2022. The median follow up for all patients is 6.5 months and the median follow up for patients still alive is 37 months. Patient characteristics are described in Table 1. The main patient related factors (sex, age, performance and co-morbidities) and disease related factors (ELN2017 risk, secondary AML, MDS and WBC) were equally distributed over both arms. In the decitabine arm (arm A) 67% were male, median age was 74 years (range 57–88), 82% had a performance score WHO 0–1 and 56% had an HCT-CI ≥ 3. In the decitabine + midostaurin arm (arm B) 59% were male, the median age was 75 years (range 55–85), 79% had a performance score WHO 0–1 and 49% had an HCT-CI ≥ 3. In arm A 34% had 2017 ELN adverse risk and 16% secondary AML; in arm B 39% had ELN2017 adverse risk and 11% secondary AML.

**Table 1:** Patient characteristics

	10-day decitabine (*N* = 70)	10-day decitabine + midostaurin (*N* = 70)
Gender: male/female	47 / 23 (67% / 33%)	41/29 (59% / 41%)
Age: median/range	74 / 57–88	75 / 55–85
WHO performance WHO 0 WHO 1 WHO 2	22 (31%) 36 (51%) 12 (17%)	21 (30%) 34 (49%) 15 (21%)
Co-morbidity: HCT-CI: 0–2 HCT-CI: ≥ 3	31 (44%) 39 (56%)	36 (51%) 34 (49%)
ELN risk group (AML) Favourable Intermediate Adverse MDS Unknown	5 (7%) 19 (27%) 24 (34%) 12 (17%) 10 (14%)	9 (13%) 16 (23%) 27 (39%) 10 (14%) 8 (11%)
Primary AML Secondary AML MDS	48 (69%) 11 (16%) 11 (16%)	50 (71%) 10 (14%) 10 (14%)
WBC (10^9^/L): median/range	3.9 / 0.70–28.4	3.2 / 0.40–29.7

*WHO* World Health Organization; *HCT-CI* hematopoietic cell transplantation Comorbidity Index; *ELN* European leukemia net; *AML* Acute myeloid leukemia; *MDS* myelodysplastic syndromes; *WBC* White Blood cells

All 70 patients allocated to arm A started 10-day decitabine, 59 patients (84%) received a second cycle and 45 patients (64%) received a third cycle. In arm B, all 70 patients started cycle 1, 51 patients (73%) received a second cycle and 35 patients (50%) received a third cycle. Main reasons to discontinue treatment are death, progressive disease/relapse and patients’ decision (Fig. 1). “Full dose” decitabine according to protocol was given in arm A in 99%, 96% and 96% of patients and in arm B in 94%, 90% and 89% in the first 3 cycles respectively. The “full dose” midostaurin was given in 49%, 43% and 57% during the first 3 cycles; and “stopped early” in 31%, 27% and 17% during the first 3 cycles; “not given” in 7%, 20% and 17% of patients during the first 3 cycles. Non haematological toxicity was the main reason for deviation in the treatment schedule of midostaurin during cycle 1, 2 and 3 (respectively 48%, 77% and 88%), while haematological toxicity was rarely the reason for deviation in the treatment schedule of midostaurin during cycle 1,2 and 3 (respectively 10%, 8% and 13%). The median number of cycles decitabine applied was 4 in arm A (range 1–33) and 3 (range 1–25) in arm B. Seven patients received an allogeneic hematopoietic cell transplantation (4 in arm A, 3 in arm B).

### Treatment outcome according to randomization

The cumulative CR/CRi rate attained during the first 3 cycles (primary study endpoint) was 24/70 (34%; 95% confidence interval (CI) 23-45%) in arm A and 17/70 (24%; 95% CI 14%-34%) in arm B and not different between the treatment arms. Some patients obtained a CR/CRi after the first 3 cycles, resulting in a final CR/CRi rate (on protocol) of 36% in arm A and 26% in arm B.

**Fig. 2:**
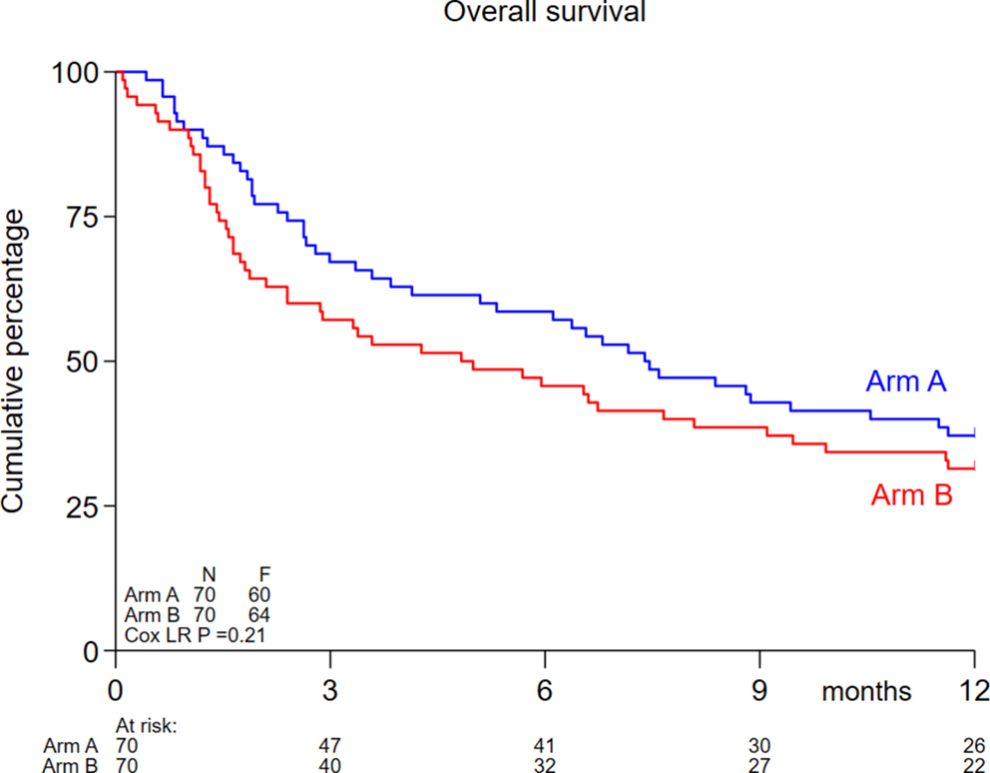
Kaplan-Meijer estimates for OS. Arm A = 10-day decitabine; arm B = 10-day decitabine plus midostaurin

At time of the final analysis in total 123 patients had died. Median OS was 7.4 months in arm A and 4.8 months in arm B. OS at 12 months was comparable between both arms (A vs. B) 37% (95% CI 26%-48%) versus 31% (95% CI 21%-42%) (Logrank *P* = 0.21) (Fig. 2).

**Fig. 3:**
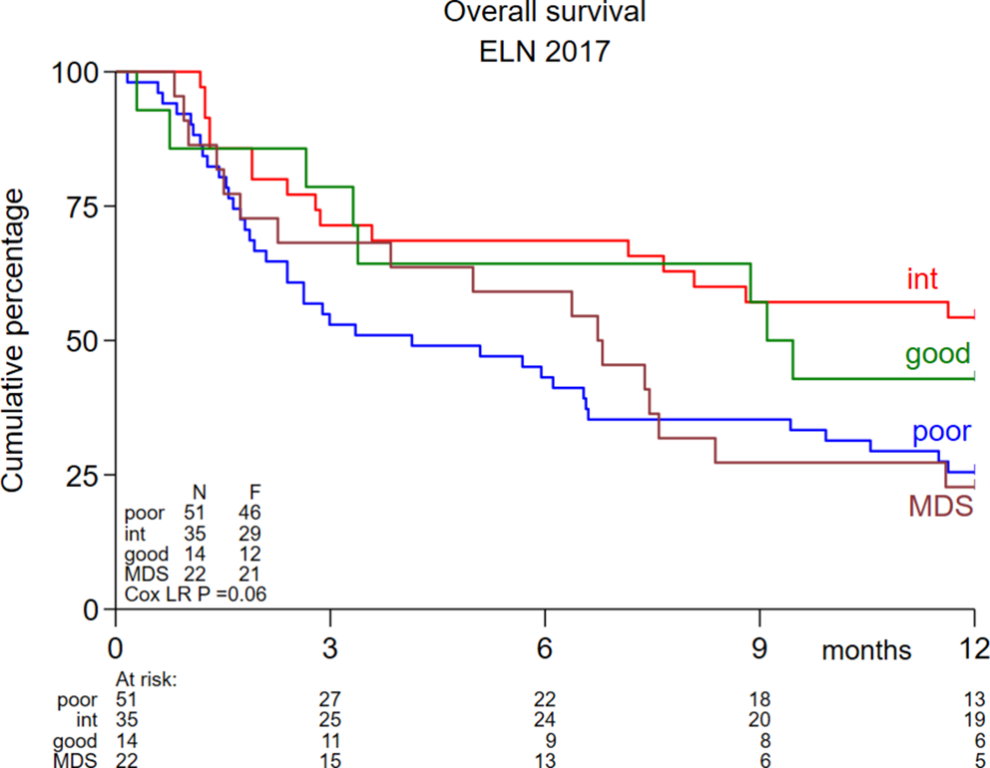
Kaplan-Meijer estimates for OS for the various ELN-2017 risk groups. *ELN* European Leukemia Net

The CR/CRi rates within the first 3 cycles were 50% for favorable, 40% for intermediate and 22% for adverse risk AML according to ELN 2017, and 18% for the higher-risk MDS patients. The OS was not different for the favorable, intermediate, adverse risk AML and higher-risk MDS subgroups (Logrank *P* = 0.06) (Fig. 3).

### Tolerability of treatment

During cycle 1 the median number of nights in hospital was 10 (range 0–30) in arm A and 12 (range 0–41) in arm B, during cycle 2 the median number of nights in hospital was 7 (range 0–39) in arm A and 1 (range 0–28) in arm B (Table 2). During the third cycle the median nights in the hospital for both arms was 0. The median number of RBC transfusions in arm A and B were comparable: 5 vs. 6, 5 vs. 4 and 2 vs. 4 respectively during cycles 1, 2 and 3. Also the median number of platelet transfusions in arm A and B was comparable: 2 vs. 3, 2 vs. 1 and 0 vs. 0 respectively during cycles 1, 2 and 3.

**Table 2:** Tolerability of treatment per treatment arm

	10-day decitabine (*N* = 70)	10-day decitabine + midostaurine (*N* = 70)
**Cycle 1** Nights in hospital (median) # RBC transfusions (median) # plt transfusions (median)	10 5 2	12 6 3
**Cycle 2** Nights in hospital (median) # RBC transfusions (median) # plt transfusions (median)	7 5 2	1 4 1
**Cycle 3** Nights in hospital (median) # RBC transfusions (median) # plt transfusions (median)	0 2 0	0 4 0
**Adverse events (AE) (any)** Grade 2 Grade 3 Grade 4	255 176 26	230 183 34
**Patients with serious adverse events (SAE)** 0 SAE 1 SAE 2 SAE 3 SAE >3 SAE	12 (17%) 24 (34%) 17 (24%) 10 (14%) 7 (10%)	10 (14%) 31 (44%) 19 (27%) 6 (9%) 4 (5%)

*RBC* Red Blood Cells; *plt* platelets

The total number of grade 2–4 AE’s was 485 (117 of which SAE) in arm A and 482 (104 of which SAE) in arm B (Table 2). The number of any CTC grade 2, grade 3 or grade 4 AE was 255, 176 and 26 in arm A and 230, 183 and 34 in arm B. Grade 3/4 infections and infestations were 14% in arm A and 11% in arm B. The early death rate (within 30 days from registration) was 10% in arm A and 10% in arm B.

## Treatment outcome according to molecular profile

Comprehensive molecular analysis at diagnosis was performed in 140 cases. The most frequently mutated genes were *DNMT3A* (24%), *TET2* (24%), *ASXL1* (24%), *SRSF2* (24%), *RUNX1* (21%) and *TP53* (17%) (Supplementary Table 1). The various mutations were generally equally distributed over both treatment groups. The response rates (CR/CRi) after 3 cycles of treatment were not different for most of the molecular abnormalities (Supplementary Table 1). The number of *FLT3-* ITD (*n* = 12) and -TKD (*n* = 10) were too small to compare both treatment arms in these genetic subgroups.

### Treatment outcome according to minimal residual disease (MRD)

**Fig. 4:**
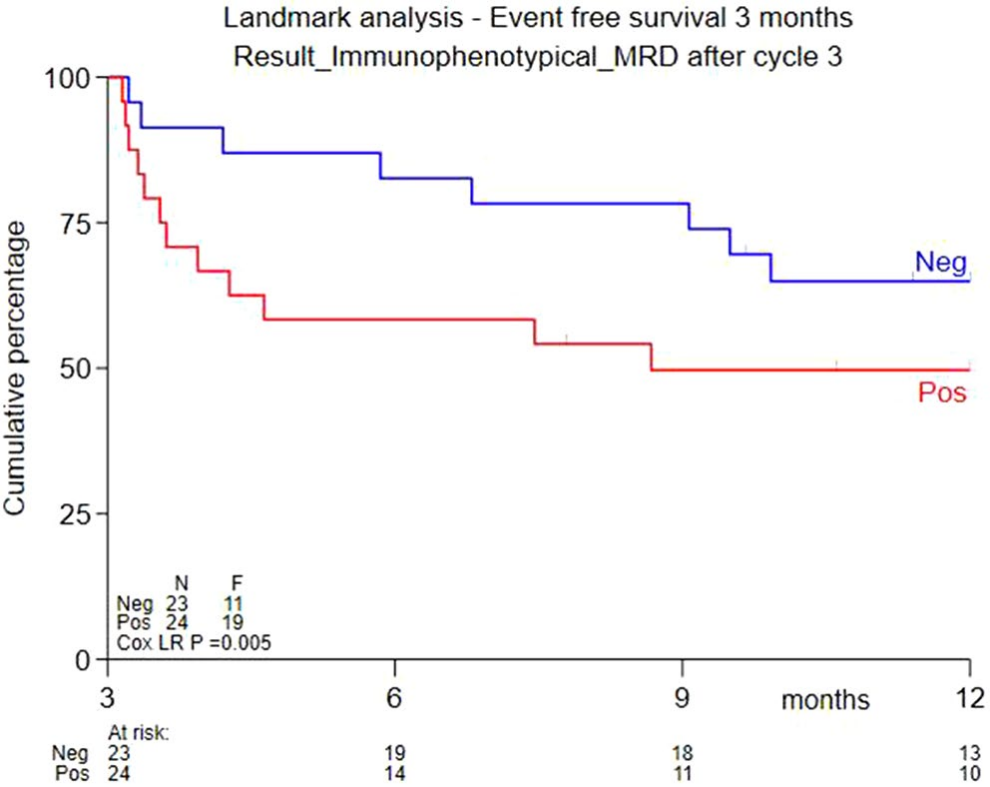
EFS estimates at 9 months MRD negative group (65%) and in the MRD positive group (50%) (p-value 0.005). *MRD* Minimal Residual Disease

Leukaemia associated immune phenotype (LAIP) was determined by multiparameter flowcytometry in all 133 patients using bone marrow samples prior to start of study treatment. MRD was assessed in bone marrow samples after cycle 3 in 58 patients. Of the 41 CR/CRi responders 26 had MRD assessments after cycle 3 of whom 17 were MRD negative. A landmark analysis after 3 months showed a superior EFS at 9 months for patients reaching MRD negativity after 3 cycles (65% vs. 50%; *P* = 0.005) (Fig. 4).

## Discussion

Hypomethylating agents (HMA) are an important treatment modality in older unfit AML and higher risk MDS patients. Here we report on the therapeutic effect of midostaurin added to a 10-day schedule of decitabine. Recent clinical insights regarding the additive inhibitory effects of midostaurin on AML blasts with mutated *FLT3* treated with intensive chemotherapy led us to embark on a randomized phase II clinical trial in older patients with newly diagnosed AML to investigate the additive impact of midostaurin on AML blasts, irrespective of their *FLT3* mutational status, treated with 10-day decitabine. This study, that compared a 10-day decitabine schedule with a combination of decitabine plus midostaurin, did not reveal a therapeutic advantage for the midostaurin combination. No improvement in response was noted for the midostaurin arm. The number of patients was too small to allow investigating a therapeutic benefit in a particular molecular subset (e.g. those with mutated *FLT3*).

The CR/CRi rate (which was the primary endpoint of the study) and the OS rate compared unfavorably with the outcome of our previous trial exploring the 10-day decitabine schedule [21], despite good adherence to the 10-decitabine application within the study. This illustrates the variability in outcome of smaller studies, and further highlights the importance of larger prospective randomized studies to draw well-founded conclusions. The current outcome data are comparable with other open label randomized trials using decitabine, which had reported median OS of 7.7 months (95% CI, 6.2–9.2) with the 5-day decitabine schedule and 9.3 months (95% CI, 5.8–12.2) with the 10 day schedule [6, 22]. Although, the application of full dose decitabine was a little bit lower in the experimental arm, only about 50% of the patients in the experimental arm received full dose midostaurin. Although detailed information is lacking, it seemed that especially non-haematological toxicity and not haematological toxicity were the main reasons for deviation of the midostaurin treatment schedule. This could be a potential explanation for the absence of a beneficial effect of addition of midostaurin to decitabine and makes it difficult to draw firm conclusions from our trial.

Like for the study reported here, other previously reported randomized trials had also failed to demonstrate a benefit for additions to HMA with histone deacetylase (HDAC) inhibitors (including entinostat, valproic acid, vorinostat, and pracinostat), the immune modulating agent lenalidomide, the proteasome inhibitor bortezomib and the Bruton’s tyrosine kinase (BTK) inhibitor ibrutinib [21–28]. The FLT3 inhibitor gilteritinib did also not improve OS when added to azacitidine in older unfit patients with AML with mutated FLT3 (LACEWING trial), despite a significantly higher complete remission rate of 58.1% versus 26.5% for the azacitidine alone arm (*P* < 0.001) [10]. Also adding midostaurin to intensive chemotherapy in fit patients with AML without *FLT3* mutation did not impact outcome [29]. So far, only venetoclax and ivosidenib have been shown to improve the HMA backbone [8, 9]. Adding venetoclax to azacitidine improved median OS from 9.6 months to 14.7 months in the whole study cohort, but the median OS of patients with adverse cytogenetics and *TP53* mutations was only 5.2 months.

In summary, here we report the final analysis of the prospective randomized phase II HOVON 155 trial, comparing a 10-day decitabine schedule with or without midostaurin, in unfit newly diagnosed *FLT3* mutation agnostic AML patients. The addition of midostaurin to 10-days of decitabine was well tolerated but did not improve remission rate nor OS.

## Electronic supplementary material

Below is the link to the electronic supplementary material.

Supplementary Material 1

## Data Availability

No datasets were generated or analysed during the current study.
