# Supplementary material for: Midostaurin added to 10-day decitabine, for patients unfit for intensive chemotherapy with AML and higher risk MDS, irrespective of FLT3 mutational status, does not improve outcome
Source: Ann Hematol. 2024 Oct 5;104(1):361–8. doi: 10.1007/s00277-024-06033-y (PMC11868317; doi:10.1007/s00277-024-06033-y)
Supplement: Supplementary file 1 — Supplementary Material 1 [file 277_2024_6033_MOESM1_ESM.docx]

**Supplementary data:**

**Methods: Molecular analyses and flowcytometry**

Bone marrow aspirations or peripheral blood samples at diagnosis were taken. Blasts and mononuclear cells at diagnosis were purified by Ficoll-Hypaque (Nygaard, Oslo, Norway) density gradient centrifugation and cryopreserved. Fresh cells were directly lysed in RLT solution with the addition of DTT (Qiagen, Venlo, The Netherlands). High quality DNA and RNA was extracted using the QIAsymphony (Qiagen, Venlo, The Netherlands). DNA concentration was measured by Qubit Fluorometric Quantitation (Thermo Fisher Scientific, Wilmington, DE) and RNA concentration using the IMPLEN Nanophotometer (Westburg, Leusden, the Netherlands). *CBFB::MYH11, RUNX1::RUNX1T1*, *FLT3* internal tandem duplications (ITD) and *FLT3* tyrosine kinase domain (TKD) mutations were determined as described previously (12, 17). Mutations in 38 out of 54 genes frequently mutated in hematologic malignancies were determined by targeted next generation sequencing (NGS) with the Illumina TruSight Myeloid Sequencing panel following the manufacturer’s protocol (Illumina, San Diego, CA). The NGS libraries were paired-end sequenced (2x221bp) on an Illumina MiSeq System (Illumina, San Diego, CA). Variants were called as described (18).

Residual disease detection by multiparameter flow cytometry (MFC) was performed as described previously (17). The residual disease percentage was defined as the number of leukemia-associated immuno phenotype (LAIP) cells within the bone marrow compartment. Based on flow cytometry, the threshold between residual and no residual disease was established and validated at 0.1% (19, 20). Information on clones, commercial sources of all monoclonal antibodies used are provided in the supplementary table 1.

**Supplementary Table 1.**

| **Gene** | **No. mutated individuals** | **No. (%) CR/CRi after 3 cycles** |
| --- | --- | --- |
| *DNMT3A* | 34 | 11 (32%) |
| *TET2* | 34 | 8 (24%) |
| *ASXL1* | 33 | 3 (9%) |
| *SRSF2* | 34 | 8 (24%) |
| *RUNX1* | 29 | 5 (17%) |
| *TP53* | 24 | 10 (42%) |
| *IDH2* | 21 | 5 (24%) |
| *NRAS* | 15 | 3 (20%) |
| *NPM1* | 22 | 8 (36%) |
| *STAG2* | 8 | 2 (25%) |
| *U2AF1* | 8 | 0 (0%) |
| *SF3B1* | 9 | 2 (22%) |
| *JAK2* | 4 | 0 (0%) |
| *BCOR* | 15 | 2 (13%) |
| *EZH2* | 9 | 2 (22%) |
| *CEBPA* | 9 | 4 (44%) |
| *FLT3* TKD | 10 | 2 (20%) |
| *FLT3* ITD | 12 | 5 (42%) |

**Supplementary table 1. Molecular abnormalities and response.** Data from 140 patients with available extensive molecular analyses are considered.
